# Deep learning models for segmenting phonocardiogram signals: a comparative study

**DOI:** 10.1371/journal.pone.0320297

**Published:** 2025-04-14

**Authors:** Hiam Alquran, Yazan Al-Issa, Mohammed Alsalatie, Shefa Tawalbeh

**Affiliations:** 1 Department of Biomedical Systems and Informatics Engineering, Yarmouk University, Irbid, Jordan; 2 Department of Computer Engineering, Yarmouk University, Irbid, Jordan; 3 Computer Engineering Department, Princess Sumaya University for Technology, Amman, Jordan; 4 The Institute of Biomedical Technology, King Hussein Medical Center, Royal Jordanian Medical Service, Amman, Jordan; 5 Department of Biomedical Engineering,Jordan University of Science and Technlogy, Ramtha, Irbid, Jordan; Instituto Nacional de Pesquisas Espaciais,BRAZIL

## Abstract

Cardiac auscultation requires the mechanical vibrations occurring on the body’s surface, which carries a range of sound frequencies. These sounds are generated by the movement and pulsation of different cardiac structures as they facilitate blood circulation. Subsequently, these sounds are identified as phonocardiogram (PCG). In this research, deep learning models, namely gated recurrent neural Network (GRU), Bidirectional-GRU, and Bi-directional long-term memory (BILSTM) are applied separately to segment four specific regions within the PCG signal, namely S1 (lub sound), the systolic region, S2 (dub sound), and the diastolic region. These models are applied to three well-known datasets: PhysioNet/Computing in Cardiology Challenge 2016, Massachusetts Institute of Technology (MITHSDB), and CirCor DigiScope Phonocardiogram.The PCG signal underwent a series of pre-processing steps, including digital filtering and empirical mode decomposition, after then deep learning algorithms were applied to achieve the highest level of segmentation accuracy. Remarkably, the proposed approach achieved an accuracy of 97.2% for the PhysioNet dataset and 96.98% for the MITHSDB dataset. Notably, this paper represents the first investigation into the segmentation process of the CirCor DigiScop dataset, achieving an accuracy of 92.5%. This study compared the performance of various deep learning models using the aforementioned datasets, demonstrating its efficiency, accuracy, and reliability as a software tool in healthcare settings.

## Introduction

For the last 20 years, Cardiovascular Diseases (CVD) have been globally the leading cause of death [1]. Taking an estimated 17.9 million lives per year [1]. In addition to mortality, CVD is responsible for long-life disabilities and it decreases the quality of life thus increasing the frequency of hospital admission. Moreover, compared with other diseases, CVD care, and treatment cost is expensive [2]. Most of the CVD-caused death cases are from low-to-middle-income countries [3]. These countries lack access to integrated healthcare systems for CVD diagnosis and treatment preventing early diagnosis and leading to further disease complications [4]. Different screening methods for CVD have been developed, such as advanced cardiac monitoring and imaging schemes (i.e., cardiac computed tomography, echocardiogram, single-photon emission computed tomography, cardiac MRI, etc.). Despite the development of previous advanced methods, cardiac auscultation remains an important first-line screening, non-invasive, and cost-effective tool.

Cardiac auscultation is the acquisition of mechanical vibrations on the body surface that convey the frequency range of heart sounds. Various cardiac structures pulsing and moving blood generate sounds that are detected and recorded as a phonocardiogram (PCG). The PCG signal provides vital clinical information for analyzing many heart abnormalities. However, training and experience are necessary for physicians to analyze such PCG signals. Moreover, the advances in medical imaging methods for CVD screening are gaining attention thus neglecting cardiac auscultation. In this regard, a motivation for computer-aided design (CAD) using PCG signals to diagnose CVDs is emerging. CAD using PCG serves as an objective tool for structural heart disease screening compared with subjective, time-consuming, and costly manual cardiac auscultation methods.

The availability of large data sets for PCG signals is a fundamental step for developing efficient CAD systems that contain rich characterization of heart sound abnormalities. In this paper we used three datasets namely: CirCor DigiScope Phonocardiogram Dataset [5], PhysioNet/Computing in Cardiology Challenge 2016 (PhysioNet) [6], and Massachusetts Institute of Technology MITHSDB [7]. They are online freely available large-scale datasets containing recordings collected from the four main auscultation locations, which contain manually annotated heart sounds. Many researchers tried to develop a CAD system that uses PCG data [8–17]. Most of this work relies on machine learning and deep learning methods. In this paper, we focus on employing state-of-the-art techniques to segment the PCG signal using a Gated Recurrent Neural Network (GRNN), Bi-Directional Gated Recurrent Neural Network (Bi-GRNN), and Bi-Long Short-Term Memory (Bi-LSTM) on the CirCor Digi Scope dataset. Furthermore, we apply our method to the PhysioNet and MITHSDB datasets to assess the generalization of the proposed model.

Specifically, the proposed method will efficiently segment PCG signals to extract the S1 and S2 heart sound waveforms and diastolic, and systolic regions. Where S1 represents the closure of the atrioventricular valve and S2 represents the closure of the semilunar valve. This paper aims to develop an automated segmentation method for the phonocardiogram signal. There are five main contributions in this paper, first, this work is the first to segment the PCG in the CirCor DigiScope dataset. Second, the accuracy of the proposed segmentation method was better than that of existing methods in the literature that were applied to the popular PhysioNet/CinC, and MITHSDB datasets. Third, this paper involves a performance comparison among three deep learning models (GRU, BIGRU, and BI-LSTM) to segment heart sounds. Fourth, the proposed model is simple and efficient as measured by the training time. Finally, this research uses signals instead of images, they require less computational complexity and storage requirements. In the related works section, an extensive literature review is conducted on recent works that developed a CAD system using PCG signal segmentation to detect heart abnormalities.

## Related works

In the realm of health informatics and computational diagnostics, recent studies have increasingly employed machine learning (ML) and deep learning (DL) techniques to enhance the accuracy, efficiency, and scalability of medical diagnostics and predictions. Literature showcases a diverse range of applications, from disease classification [18–20] to predicting physiological characteristics [21–23]. and optimizing healthcare outcomes [19,24]. However, here we will focus on related works that cross with our methodology and objective, highlighting the overlaps, pros, and cons of our work. In 2018, Messner et al. [25]. introduced a novel approach for detecting the state sequence of heart sounds S1 and S2 using a deep recurrent neural network (DRNN). Their methodology involved extracting spectral and envelope features from the recordings and applying dropout for regularization. They also evaluated the performance of various DRNN architectures in accurately identifying the state sequence. In this paper a model that uses frequency domain analysis as in Messner et al. was built, However, it also uses time series data which resulted in a reduction in computational complexity. Moreover, Chen et al. [26], presented a novel end-to-end method based on convolutional long short-term memory (CLSTM), which directly uses audio recording as input to address the Heart Sound Segmentation (HSS) task. The convolutional layers were designed to extract the meaningful features and perform the down sampling, and the LSTM layers were added to perform the sequence recognition. Both components collectively improve the robustness and adaptability of the processing of the HSS task. However, the complexity of the layer design was one drawback to their design; they used Bi-LSTM due to its flexibility since it can adapt to various signal lengths, not like convLSTM which is limited to fixed-size images or video sequences. Chen et al. [27] used LSTM for heart sound segmentation to detect heart valve diseases. They proposed a duration LSTM network to effectively model the intrinsic sequential characteristic by incorporating the duration feature. The proposed method performed well on different tolerance windows. Fan et al. [28] proposed a direct heart sound segmentation approach using bidirectional gated RU and time-frequency-based features. They presented 96.86% accuracy for four class classifications. However, their proposed methods of analysis lack precision, as mentioned in the reference above. Fernando et al. [29], used wavelet transform MEL frequency (MFCC), Bi-LSTM, and attention-based deep learning mechanism to segment heart sounds. Finally, Xu et al. [30] compared different deep learning structures, and they proposed a method that uses (Self-Modulating Gating Unit) SMGU-RNN and achieved an accuracy of 88%. In addition, they used Wavelet transform SMGU-LSTM which increased the computational complexity of their algorithm. In our approach, the GRU and gated LSTM we used are less complex and easy to train. In this paper, sequential deep learning models are applied for heart sound segmentation. The datasets used in this paper are the CirCor DigiScope Phonocardiogram Dataset [5], PhysioNet [31], and MITHSDB [7]. Furthermore, this paper contains a performance comparison among three deep learning models (GRU, BIGRU, and BI-LSTM) to segment heart sounds using the previously mentioned datasets.

## Materials and methods

**Fig 1:**
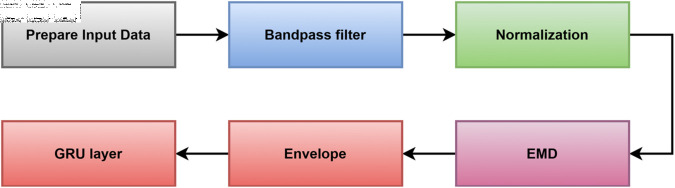
The block diagram for the proposed method.

The main goal behind this paper is to build an automated system to localize the heart sound wave segments, S1, S2, systolic, and diastolic regions. Fig 1 describes the proposed method. The corresponding subsections explain each block separately.

### Data preparation

In this study, various PCG datasets have been used:

1. The PhysioNet/Computing in Cardiology Challenge 2016, This data was gathered over a period of more than ten years from nine heart sound databases that were amassed independently by seven distinct research teams. A total of 3153 recordings with 233,512 heart sounds were made from 116,865 heartbeats from 1,072 patients [31].
2. MITHSDB contains the heart sound database for the Massachusetts Institute of Technology (MITHSDB) by Dr. Zeeshan. A total of 121 participants provided 409 PCG recordings from nine different locations and orientations. Each subject provided multiple recordings. Notably, this data is part of the PhysioNet Challenge database.
3. The CirCor DigiScope Phonocardiogram Dataset, 2916 heart sound recordings totaling over 33.5 hours of recording have been collected from the major four auscultation positions of 942 subjects. A human annotator has provided in-depth annotations for each heart murmur in the dataset. Additionally, a semi-supervised method was used to derive segmentation annotations on the location of the basic heart sounds (both S1 and S2) in the recordings [5]. The stethoscope is ideally placed at specific locations: The Aortic Valve (AV) which is the second intercostal space, right sternal border, Pulmonic Valve (PV) which is the second intercostal space, left sternal border where the Tricuspid Valve (TV) is the left lower sternal border, and the Mitral Valve (MV): fifth intercostal space, midclavicular line (cardiac apex). The recording was measured from various valves (Aortic, Pulmonic, Tricuspid, and Mitral values). The number of recordings for each site is illustrated in Table 1, and the total number of recordings is 2919. To decrease training time and improve segmentation accuracy, the signal was divided into many segments, each lasting 2 seconds.

**Table 1:** The distribution of recording for four heart valves

Type	Number of recordings	Number of segments
Aortic valve	723	3482
Pulmonic valve	712	4737
Tricuspid valve	687	4568
Mitral valve	794	4338
Any other auscultation location	3	–
All type	2919	17125

Each PCG signal file contains the variable heart signal and a table of signal region labels limit (ROI). Each file also contains the sample rate of the signal in variable frequencies. All signals have a sample rate of 4000 Hz. Table 2 represents an example of one recording describing the region label.

**Table 2:** The signal region label

ROI limits		Class
1	555	“S1”
555	1035	“systolic”
1035	1515	“S2”
1515	2235	“diastolic”
2235	2795	“S1”
2795	3275	“systolic”
3275	3755	“S2”
3755	4555	“diastolic”

**Fig 2:**
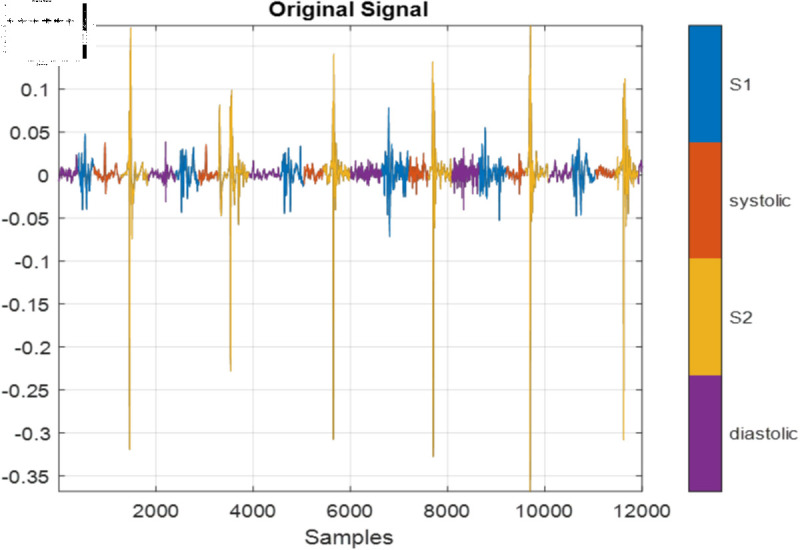
The PCG signal for one recording.

For the proposed work, the region labels on the dataset must be transformed to sequences containing one label per signal sample. Fig 2 shows the labeled PCG signal for one recording.

### Signal pre-processing

In this section, signal pre-processing steps will be introduced in detail.

#### BandPass filter.

The PCG signal is filtered by passing it to a 4th order bandpass Butterworth filter with a lower cutoff frequency of 60 Hz and a high cutoff frequency of 150 Hz.

#### Normalization.

The filtered signal is normalized to be in the [–1,+1] range. Using the following equation:

$$\begin{array}{ccc} x_{\text{normalized }}=\frac{\left(x-x_{\min}\right)}{\left(x_{\max}-x_{\min}\right)} & & \end{array}$$ (1)

where *x* represents the PCG signal.

#### Empirical mode decomposition.

The normalized signal is further processed by Empirical Mode Decomposition (EMD) techniques, which were proposed by Huang in 1998 [32]. EMD is a technique for analyzing non-stationary signals and overcoming the problems of the availability of base functions as wavelet and Fourier transforms. It is focused on decomposing the signal into various oscillatory AM-AF levels each one is called intrinsic empirical mode function (IMF) using an iterative procedure (namely sifting procedure). Every Intrinsic Mode Function (IMF) is required to satisfy two distinct criteria: firstly, it must exhibit an equivalence in the count of extrema (including maxima and minima) and zero crossings, with a permissible difference of at most 1. Second, the average value of the envelopes derived from local maxima and minima is (approximately) zero. The procedure for applying EMD is as follows [33]:

Determine all the maxima and minima points of *x*(*n*).
Do spline interpolation between maxima and minima points to obtain a lower envelope and upper envelope. Calculates the mean of the obtained two envelopes using following equation
  $$\begin{array}{ccc} x_{n}=\frac{\left(lower\;envelope+upper\;envelope\right)}{2} & & \end{array}$$ (2)
(c) Substrate the resultant mean from the original signal. If the difference does not meet the IMF conditions then the iterative procedure is stopped, otherwise, the iteration continues.
  $$\begin{array}{ccc} d(n)=x(n)-m(n) & & \end{array}$$ (3)
(d) If *d*(*n*) meets the criteria of IMF, then it counts as IMF, and another signal is obtained by subtracting the original signal from IMF, which is called the residual signal
  $$\begin{array}{ccc} r(n)=x(n)-d(n) & & \end{array}$$ (4)
(e) Perform the previous steps from 1–5 for the residual signal until a monotonic or constant signal is obtained. The following equation explains how the original signal is constructed from the decomposed level:
  $$\begin{array}{ccc} x(n)=\overset{L-1}{\underset{i=1}{\sum }}IMF^{i}+r{\left(n\right)}^{L} & & \end{array}$$ (5)
  where L represents the decomposition level, and i refers to the IMF order. In this paper, the number of decomposed levels is 3, for the proposed method we choose the first intrinsic mode function. The first IMF is excluded for further processing.

#### Envelope.

The envelope is performed on the first IMF bypassing the analytical signal to the Finite impulse response filter with a length of 200. The analytic signal can be represented by the corresponding equation

$$\begin{array}{ccc} d=x_{\text{r}}-jx_{\text{i}} & & \end{array}$$ (6)

**Fig 3:**
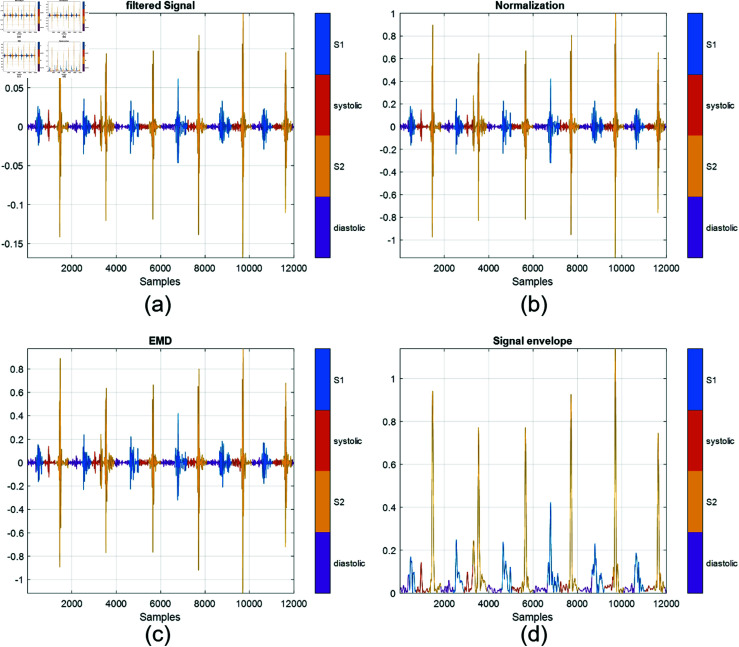
The pre-processing stages. (a) The output of bandpass filter. (b) The normalized output of filtered signal. (c) First IMF of the PCG signal. (d) The envelope of the first.

The analytical signal is the summation of the original signal and the Hilbert transform of the original signal, which forms the imaginary part of the analytical signal [34]. The Hilbert transform alters the phase of all frequency components in the original signal by 90${}^{\circ }$, resulting in an analytical signal. Another distinction between analytical signals and the original ones lies in the Fourier transform: while the original signal includes both positive and negative frequency components, the analytical signal excludes the negative frequencies. Furthermore, the original signal assigns an amplitude to each time point. In contrast, the analytical representation creates a rotating vector in the complex plane, offering instantaneous values for parameters like amplitude, power, and phase with a single sample from a discrete signal. Fig 3 explains the preprocessing steps for all stages (a) the filtration, (b) normalization, (c) first IMF, and (d) the envelope of the first IMF. The resultant output is ready for passing into the designed deep learning models.

All types of datasets undergo the same process, starting with filtration followed by the application of Empirical Mode Decomposition. The novelty of this work lies in developing a general model capable of accounting for all variations across any dataset. The proposed procedure is universal and can be applied to any dataset.

### Deep learning models

The prepared signal is passed into the designed deep learning models. Each model is trained and tested on the datasets separately, the corresponding sections depict the design and training process for each model on the prepared data.

#### Gated recurrent network.

**Fig 4:**
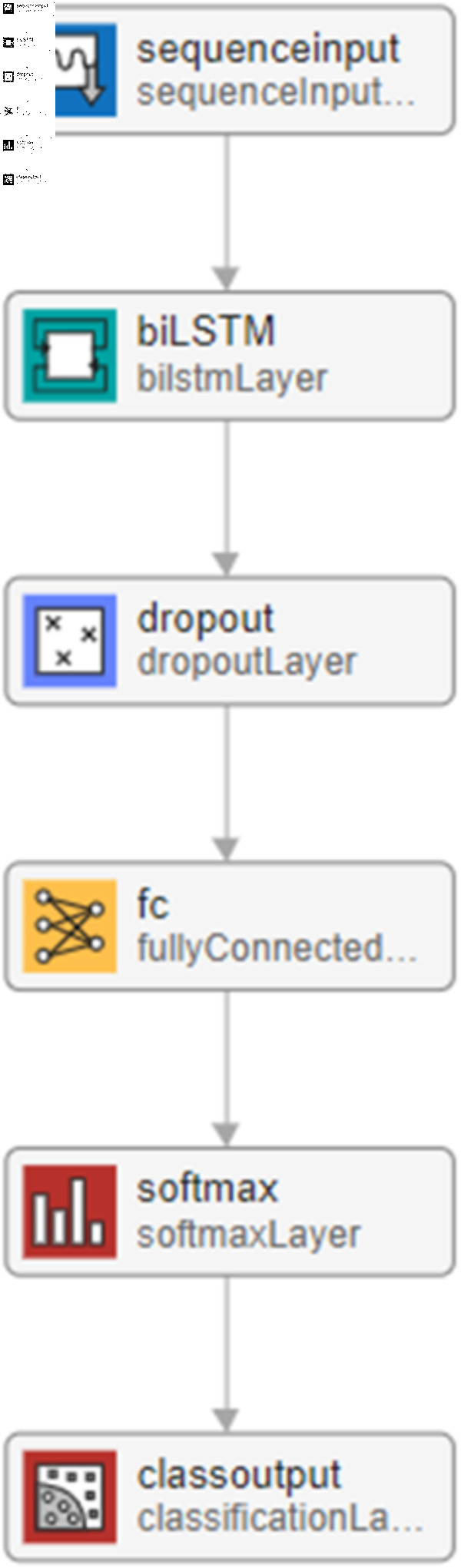
The proposed GRU network.

The proposed GRU network architecture is shown in Fig 4 which is started by the sequential layer, which is responsible for passing the time series data into the flowing GRU layer.

**Fig 5:**
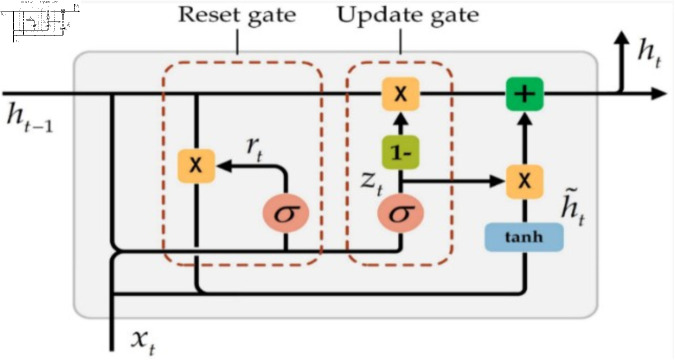
Internal structure of GRU [35].

The GRU (Gated Recurrent Unit) layer consists of several GRUs that are working sequentially. Each GRU is a type of recurrent neural network. The GRU structure combines the hidden state and cell state into one state. Moreover, it is distinguished by adaptive controlling dependencies on various time scales by selecting the keeping past and forgetting information. Because of the simplicity of its structure, it is fast in training when compared with long short-term memory (LSTM). GRU’s internal structure consists of two types of gates; the reset gate is similar to the forget gate in LSTM, and the update gate. Fig 5 illustrates the principal operation of the GRU network.

The following is the explanation of the internal structure variables [36]: $x_{t}$ is the current input, $z_{t}$ is the update gate, $r_{t}$ is the reset gate, $\tilde{h_{t}}$ is the candidate hidden state of the currently hidden node, $h_{t}$ is the current hidden state, $h_{t-1}$ is the hidden state of the previous layer. *σ* is the Sigmoid function which transfers input between 0 and 1. $\tilde{h_{t}}$ records all important information through the reset gate and inputs information

$$\begin{array}{ccc} z_{t}=\sigma \left(w_{zx}x_{t}+u_{zh}h_{t-1}\right) & & \end{array}$$ (7)

$$\begin{array}{ccc} r_{t}=\sigma \left(w_{rx}x_{t}+u_{rh}h_{t-1}\right) & & \end{array}$$ (8)

$$\begin{array}{ccc} \tilde{h_{t}}=\tanh\left(w_{hx}x_{t}+r_{t}⊙u_{hh}h_{t-1}\right) & & \end{array}$$ (9)

$$\begin{array}{ccc} h_{t}=\left(1-z_{t}\right)⊙\tilde{h}+z_{t}⊙h_{t-1} & & \end{array}$$ (10)

where *w* and *u* are the weighted vectors, their values are updated during the learning phase. The reset gate specifies the capability of a hidden state in the previous time to forget based on the content of the current time that will lead to adding the current input and passing it to the sigmoid activation function. Therefore, the important information of the current time input is stored in $h_{t}$. The important information is stored at $h_{t-1}$, which represents the hidden state of the previous layer. The GRU layer is responsible for learning dependencies between time steps in time series and sequence data. The number of units is a hyperparameter; in the proposed model it is 200.

The output of the GRU layer is a sequence that predicts class labels, the proposed network ends with a fully connected layer, a SoftMax layer, and a classification output layer. Moreover, the fully connected layer combines all of the features learned by the GRU layer for the sequences to identify the larger patterns. Therefore, the number of neurons in fully connected layers equals the number of classes. In the proposed network, there are four classes, S1, S2, systolic region, and diastolic region. SoftMax layer applies the SoftMax function on the four output features from the previous fully connected layer [37]. The following equation shows the SoftMax function:

$$\begin{array}{ccc} f\left(x_{i}\right)=\frac{e^{x_{i}}}{\underset{j}{\sum }e^{x_{j}}} & & \end{array}$$ (11)

where *x* is the input feature of size k, in our case, we have 4. The output of the SoftMax function ranges between 0 and 1. Moreover, the output is interpreted as a probability. The probability output can be interpreted as a class label in the classification layer. The training operation is performed using 70% of the whole records with an initial learning rate of 0.001, and Adaptive Moment Estimation (Adam) optimizer, 300 epochs with 16 records as mini-batch size.

#### Bi-directional gated recurrent neural network.

BI-GRU models are employed in this study. It consists of two GRU models which work in two opposite directions. One GRU is responsible for moving in the forward path (from the beginning of the sequence to the end), whereas the other oversees the backward track (which starts from the end to the origin). This enhances the prediction by considering the impact of the future and the past information on the current state. The corresponding equations clarify the two opposite ways.

$$\begin{array}{ccc} \vec{h_{t}}=GRU_{fwd}\left(x_{t},\vec{h_{t-1}}\right) & & \end{array}$$ (12)

$$\begin{array}{ccc} \overset{\leftarrow }{h_{t}}=GRU_{bwd}\left(x_{t},\overset{\leftarrow }{h_{t-1}}\right) & & \end{array}$$ (13)

**Fig 6:**
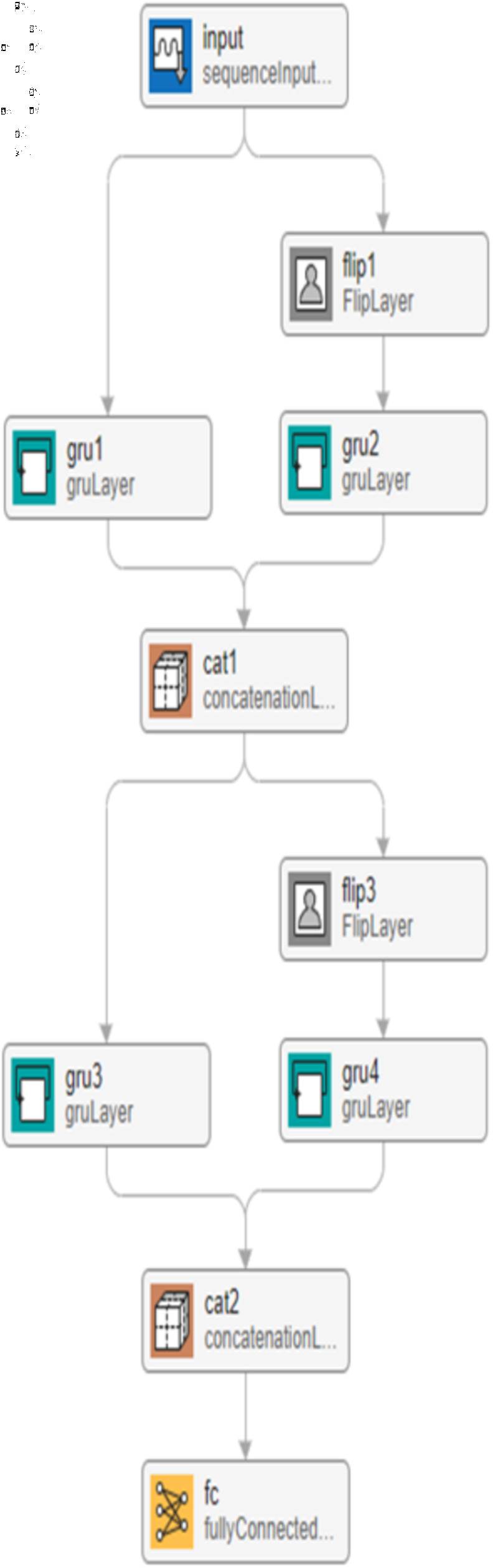
The proposed Bi-GRU network.


$$\begin{array}{ccc} h_{t}=\vec{h_{t}}⊕\overset{\leftarrow }{h_{t}} & & \end{array}$$ (14)

where $\vec{h_{t}}$ is is the state of the forward GRU , $\overset{\leftarrow }{h_{t}}$ is the state of the backward GRU and  ⊕  indicates the operation of concatenating two vectors [38]. The structure in Fig 6 has been used except the GRU layer is replaced by Bi-GRU. The network is trained with an Adam optimizer, 200 epochs, and a minibatch size of 16.The corresponding figure illustrates the proposed Bi-GRU model.

#### Bi-long short term memory (Bi-LSTM).

**Fig 7:**
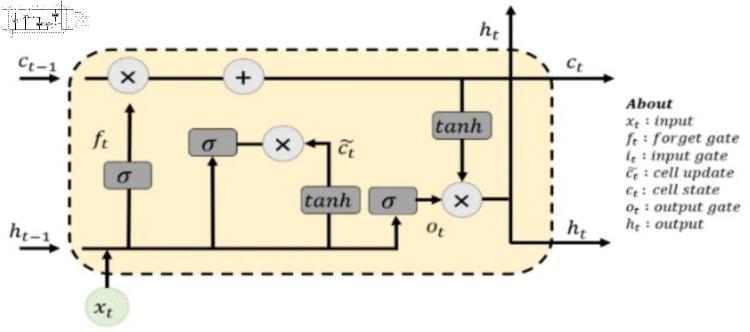
Internal structure of LSTM unit [35].

The long short-term memory cell is compromised by many components to control the flow of information over time [35,36]. Fig 7 illustrates the main components of the LSTM unit or cell.

C_t_ represents the cell state that is responsible for storing or discarding information over a long sequence. H_t_ donates to the output of LSTM units which depends on the cell state and the input. i_t_ signifies the input gate which reveals which information from the current time input should be added to the cell state. Its values are either 0 or 1 because it uses the sigmoid activation function. f_t_ indicates the forget gate, which utilizes a *sigmoid* function to which information should be discarded. O_t_ symbolizes the output gate which controls which parts of the cell state are used to produce the hidden state for the current time step using *sigmoid* and *tanh* activation functions to produce 0,1 or -1,1, as output, respectively. C~_t_ refers to the candidate gate. It is in charge of the new information that could be added to the cell state. It is calculated based on the current input and a *tanh* activation function. The LSTM unit combines the information from the input gate, forget gate, and output gate to update the cell state and hidden state at each time step [39].

The LSTM layer is composed of multiple LSTM units that are stacked together to process sequential data by updating its internal cell state and hidden state at each time step. In contrast, the Bi-LSTM layer consists of two LSTM layers, each one works from the beginning to the end, and the other one works in the opposite direction (from the end to the beginning). In this work, the network in Figure 4 has been used except the GRU layer is replaced by the Bi-LSTM layer with 200 units. The proposed network is trained with Adam optimizer and 16 minibatch size. The hyper parameters, including the number of epochs, batch size, learning rate, and optimizer, are tuned to obtain the best model during training. The best model is then tested on the remaining data.

## Results and discussion

In this section, the effectiveness of the proposed Bi-GRU model in segmenting phonocardiogram recordings is evaluated using popular datasets. Tables 3–5 compare the performance of three models GRU, Bi-LSTM, and Bi-GRU respectively. As shown in Table 5 the Bi-GRU model outperforms all other models, it is also clear from the table that segmentation works the best at MV, followed by TV, PV, and AV locations. For the Bi-GRU, the accuracy was 92.5%, sensitivity was 91.7%, specificity was 97.4%, precision was 91.9%, and F1-score was 91.8%. For the Bi-LSTM, the accuracy was 88.5%, sensitivity was 86.7%, specificity was 95.9%, precision was 89.1%, and F1-score was 87.7%. Finally, for the GRU, the accuracy was 86.3%, sensitivity was 84.2%, specificity was 95.1%, precision was 86.9%, and F1-score was 85.3%.

**Table 3:** Performance metrics for the GRU at four different body positions

Location	GRU				
	**Acc**	**Se**	**P+**	**Sp**	**F1**
*TV*	86.70%	84.60%	87.20%	95.20%	85.70%
*MV*	88.90%	88.40%	88.10%	96.30%	88.20%
*PV*	80.00%	77.70%	80.10%	92.80%	78.70%
*AV*	77.10%	75.00%	76.30%	91.90%	75.60%
*ALL*	86.30%	84.20%	86.90%	95.10%	85.30%

**Table 4:** Performance metrics for the Bi-LSTM at four different body positions

Location	GRU				
	**Acc**	**Se**	**P+**	**Sp**	**F1**
*TV*	89.70%	88.00%	90.10%	96.30%	88.90%
*MV*	91.40%	90.80%	90.80%	97.10%	90.80%
*PV*	82.80%	80.70%	83.20%	93.90%	81.80%
*AV*	80.50%	78.60%	79.80%	93.10%	79.10%
*ALL*	88.50%	86.70%	89.10%	95.90%	87.70%

**Table 5:** Performance metrics for the Bi-GRU at four different body positions

Location	GRU				
	**Acc**	**Se**	**P+**	**Sp**	**F1**
*TV*	95.10%	94.30%	95.40%	98.20%	94.80%
*MV*	96.80%	96.50%	96.60%	98.90%	96.50%
*PV*	86.80%	85.20%	87.10%	95.30%	86.00%
*AV*	81.70%	80.00%	81.00%	93.60%	80.50%
*ALL*	92.50%	91.70%	91.90%	97.40%	91.80%

**Table 6:** Performance of the Bi-GRU model using three different datasets

Dataset		Acc	Se	P+	Sp	F1
*PhysioNet* ∕ *CinC*		97.30%	96.90%	96.50%	99.10%	96.70%
*MITHSDB*		96.98%	95.96%	96.19%	98.92%	96.07%
*CirCor* *DigiScope*	*TV*	95.10%	94.30%	95.40%	98.20%	94.80%
	*MV*	96.80%	96.50%	96.60%	98.90%	96.50%
	*PV*	86.80%	85.20%	87.10%	95.30%	86.00%
	*AV*	81.70%	80.00%	81.00%	93.60%	80.50%
	*ALL*	92.50%	91.70%	91.90%	97.40%	91.80%

Table 6 compares the performance of the Bi-GRU model using three different datasets. The table shows that the accuracy using the PhysioNet/CinC dataset was 97.3%, and the accuracy using the MITHSDB dataset which is a subset of the PhysioNet/CinC dataset was 96.98%. Finally, the accuracy in the CirCor DigiScope dataset was 92.5%.

Table 7 compares the training time of the Bi-GRU model using the three different datasets. It took 7234 minutes to train the PhysioNet/CinC dataset, and 1745 minutes to train the MITHSDB dataset. Finally, in the CirCor DigiScope dataset, the training time was 1337, 1452, 1626, and 1024 minutes for TV, MV, PV, and AV locations respectively.

**Table 7:** The training time of the BI-GRU model using three different datasets

Dataset		Training accuracy	Training loss	Training time (minutes)
*PhysioNet* ∕ *CinC*		98.96%	8.27%	7234
*MITHSDB*		97.57%	9.07%	1745
*CirCor* *DigiScope*	*TV*	96.23%	15.42%	1337
	*MV*	97.65%	12.74%	1452
	*PV*	88.27%	23.40%	1626
	*AV*	85.23%	42.27%	1024

**Table 8:** Performance comparisons of the suggested model and recent literature

Ref.	Dataset	Method	# of records	Performance
Chen et al. [27]	MITHSDB	LSTM network	409	Acc: 96.86%
Messner et al. [25]	2016 PhysioNet/CinC Challenge	GRU	2874	F1: 93%
		BiLSTM		F1: 94.1%
		BiGRU		F1: 96.1%
Chen et al. [26]	MITHSDB	CLSTM	405	F1: 96.18
Fan et al. [28]	2016 PhysioNet/CinC Challenge	BiGRU	3153	Acc: 96.86% F1: 98.40%
Fernando et al. [29]	PhysioNet/ CinC Challenge 2016 (PCC)	MFCC	2874	Acc: 97.1% F1: 94.70%
	M3dicine Human HS Database (M3-Hu)	Bi-LSMT	170	
	M3dicine Animal HS Database (M3-An)	deep learning	105	
Xu et al. [30]	2016 PhysioNet/CinC Challenge	A simpler gated unit	-	Acc: 88%
*ProposedModel*	**2016 PhysioNet/CinC Challenge**	**BiGRU**	**2874**	**Acc: 97.3%**
	**MITHSDB**		**409**	**Acc: 96.98%**
	**CirCor DigiScope**		**2919**	**Acc: 92.5%**

Table 8 summarizes recent literature that experimented with PCG segmentation. The table compares the performance of the suggested model to the state of the art. The proposed model accuracy using the PhysioNet/CinC challenge dataset was 97.3% higher than the 97.1% reported by Fernando et. al. in 2019. In addition, the proposed model accuracy using the MITHSDB dataset was 96.98% which is slightly higher than the 96.86% reported by Chen et. al in 2020. Finally, the proposed model achieved 92.5% accuracy using the CirCor DigiScope dataset which is the largest dataset used (2919 recordings). This paper was the first to report the accuracy of the CirCor DigiScope dataset which contains the largest number of records among all known datasets. In addition, the simplicity of the proposed network makes it easy to implement and run with impressive results. All previous studies employed various advanced signal processing techniques to extract the most relevant features and passed these features as input to LSTM, GRU, or Bi-LSTM. Although the previous studies used sophisticated tactics, the simple model discussed in this paper achieved higher accuracy than what was previously reported. The current approach can be further improved by adding more features to the LSTM.

**Fig 8:**
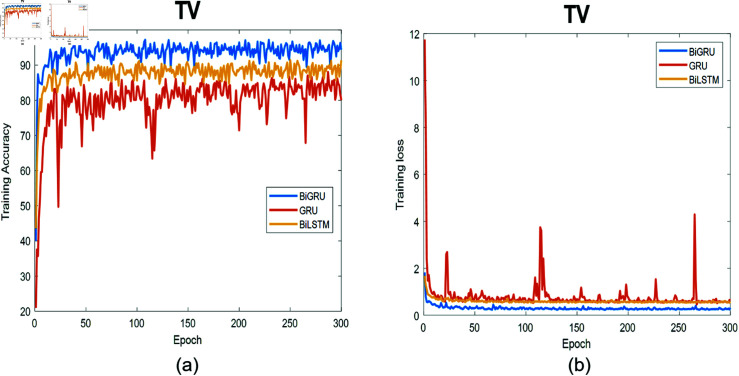
The training process for TV data. (a) Training accuracy as a function of epochs. (b) Training loss as a function of epochs.

**Fig 9:**
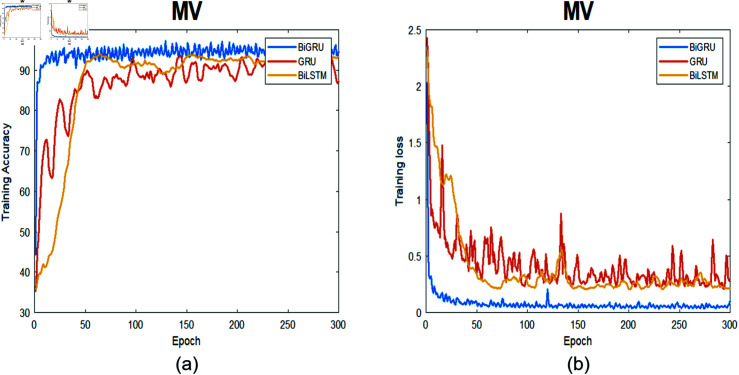
The training process for MV data. (a) Training accuracy as a function of epochs. (b) Training loss as a function of epochs.

**Fig 10:**
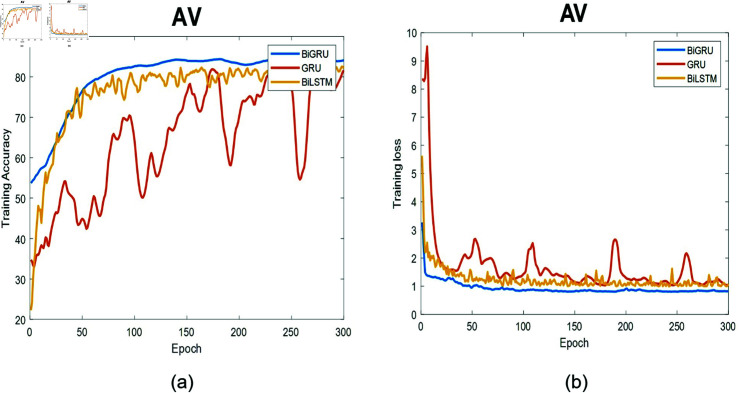
The training process for AV data. (a) Training accuracy as a function of epochs. (b) Training loss as a function of epochs.

**Fig 11:**
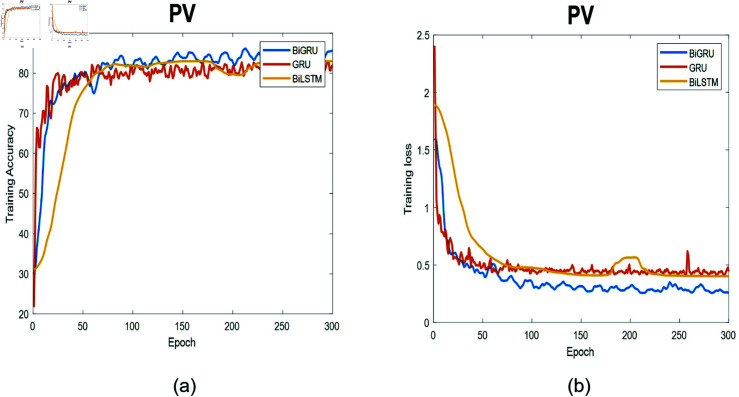
The training process for PV data. (a) Training accuracy as a function of epochs. (b) Training loss as a function of epochs.

Fig 8- 11 depict the training process in terms of accuracy and training loss using the three proposed networks separately. Fig 8a describes the training process for the three deep learning models at the TV location together with the corresponding training loss for 300 epochs. The Bi-GRU model achieved the best performance followed by Bi-LSTM and then GRU. The training accuracy using BiGRU is 95%, on the other hand, the Bi-LSTM trained with 90% accuracy, whereas, GRU had the worst accuracy, and it is clear from the loss function that it did not converge.

The smoothness that both the Bi-LSTM and Bi-GRU exhibited arise from the nature of the bidirectional learning process, which enhances accuracy compared to the unidirectional learning process used in the GRU. The figure also illustrates that the GRU struggled to capture the correct underlying signal pattern as training progressed. This is evident from the observed overshooting in the training and loss curves during the learning phase.

Fig 9a illustrates the training process of the three deep-learning models at the MV location and the corresponding training loss for 300 epochs. The performance of Bi-GRU was the best followed by Bi-LSTM then GRU. The GRU had the worst behavior with fluctuations in the loss function. Among all the curves, the Bi-GRU curve exhibited the highest level of smoothness, showcasing an improved accuracy right from the start of the learning phase. In contrast, Bi-LSTM and GRU struggled to perform well at this valve. Bi-LSTM requires an extended period to grasp the underlying data patterns. Conversely, the figures affirm Bi-GRU’s ability to rapidly learn data patterns in the initial stages of the learning process.

Fig 10 demonstrates the training and loss function for three deep-learning models at the AV location. The accuracy is the worst among all locations, but still, the Bi-GRU outperforms all deep models. This difference can be attributed to the data’s inherent complexity, or it might indicate that the Bi-GRU model requires more time to precisely grasp the underlying patterns. Despite its performance not reaching a high level, Bi-GRU still demonstrates the most effective ability to differentiate between the four segments. Furthermore, the figures confirm the weakness of the GRU in learning the patterns within the available data.

Fig 11 depicts the training process for the three models at the PV location. The accuracy is one of the worst among all four valves, but the performance of the Bi-GRU continues to be the best among all surveyed models. The Bi-GRU learns quickly and outperforms all other models in effectively distinguishing between different segment types. For the PV location, all models exhibit relatively smooth training curves, but their accuracy remains comparatively low when compared to the MV location. This lower accuracy could be attributed to the inherent characteristics of the data.

**Fig 12:**
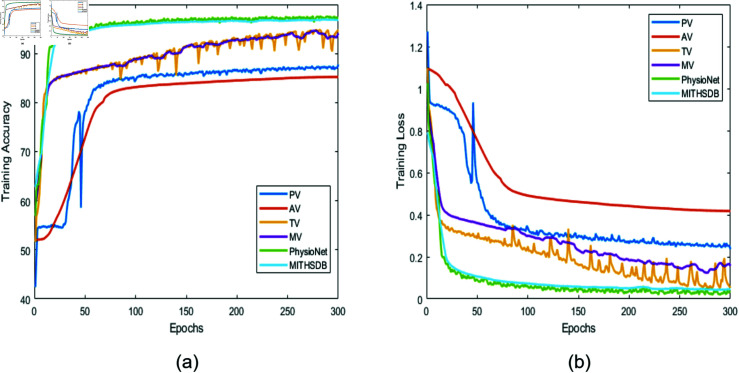
Training accuracy/loss as a function of epochs for different datasets.

Fig 12 illustrates the training accuracy for all datasets that have been used in this study using the Bi-GRU model. The figure illustrates the superior results Bi-GRU achieved across all trained datasets. In terms of accuracy, the rankings from highest to lowest were as follows: PhysioNet, MITHSDB, MV, TV, PV, and AV. As discussed earlier, these outcomes can be attributed to the combination of the data’s inherent characteristics and Bi-GRU’s ability to learn bidirectionally along with its simpler internal structure. This simplicity yields an efficient training process, as it involves fewer parameters and reduces the risk of overfitting when compared to the Bi-LSTM.

One limitation of the proposed approach is the computation time, which can be mitigated by utilizing high-performance computers to expedite the training process. It is possible that developing a model with additional Bi-GRU layers could improve performance, but this would necessitate access to high-performance computing resources. This limitation can be addressed by optimizing the computational environment, ultimately enabling the method to be adapted for real-time signal processing applications.

## Conclusion

This paper utilized deep learning models to segment phonocardiogram (PCG) signals. The process began with the application of various signal processing techniques to prepare the signals for subsequent analysis. Three different models, namely GRU, BIGRU, and Bi-LSTM, were employed to segment PCG signals using three distinct datasets PhysioNet, MITHSDB, and CirCor DigiScop, each containing heart sound data from four different valve locations. Notably, the BiGRU model outperformed the other models across all datasets, achieving the highest accuracy. The proposed model also delivered improved accuracy values for both the PhysioNet and MITHSDB datasets. Additionally, this study marked the first exploration of segmenting the CirCor DigiScop dataset, achieving an impressive accuracy of 92.6%. The simplicity, efficiency, and accuracy of the proposed models make them effective tools for distinguishing between four types of PCG segments, including S1, systolic region, S2, and diastolic region. Furthermore, the proposed approach surpasses prior studies in terms of accuracy, sensitivity, precision, and F1-score. Ultimately, this system facilitates the localization of PCG segments simplifying the diagnosis and analysis process. The research can be expanded to classify heart sounds into different categories of normalcy and abnormalities, aiming to develop a comprehensive system that begins with signal acquisition, followed by enhancement, segmentation, and classification. This would assess the severity of the condition and contribute to enhance medical diagnosis in health centers.
